# Assistance and Delivery of COVID-19 Vaccinations to Patients with Rare Diseases

**DOI:** 10.3390/healthcare11202781

**Published:** 2023-10-20

**Authors:** Mariana Carvalho de Moraes, Ivone Duarte, Rui Nunes

**Affiliations:** 1 Doctoral Program in Bioethics, Faculty of Medicine, University of Porto, 4200-319 Porto, Portugal; 2 Department of Social Sciences and Health, Faculty of Medicine, University of Porto, 4200-319 Porto, Portugal; iduarte@med.up.pt (I.D.); ruinunes@med.up.pt (R.N.)

**Keywords:** rare disease, COVID-19, vaccination, public policy

## Abstract

The COVID-19 vaccination campaign has been fortified by the positive effects of immunization: data from official government organizations and communication channels show that infection rates, hospitalizations, and deaths have continued to fall since its implementation. In Brazil, the effective and free National Vaccination Program has failed to prioritize patients with rare diseases, who have extreme comorbidities, and who adhere to the calendar prepared by the government. The question is why these “rare people” were not considered priorities during the vaccination program. This study aims to understand the reasoning behind this decision and to resume the debate around the rights of patients with rare diseases based on regular descriptors issued by official government agencies and by critics of rare disease issues in scientific articles. The objectives of this study were as follows: to analyze the dispensed care of rare disease patients in the COVID-19 vaccination campaign; to identify the procedures implemented by the National Vaccination Plan for the Brazilian population over 18 years of age and to evaluate the effectiveness of the plan implemented from the perspective of a “rare person”.

## 1. Introduction

For decades, vaccination has been the most efficient method of prevention, mitigation, and even the extinction of pathogens that can create bothersome sequelae and may lead to death.

Since the emergence of the novel coronavirus, whose severity and lethality date back to records from previous centuries, the medical and scientific society has been united in the creation of vaccines that mitigate symptomatology. COVID-19 is an acute respiratory disease caused by SARS-CoV-2 with numerous mutations, and treating it has led to new studies and new and/or constant processes of immunization.

In the Brazilian National Immunization Program (PNI), which has been referenced worldwide due to its effectiveness and free, universal coverage, rare disease patients—people with extreme comorbidities—did not receive due deference in the vaccination schedule which started in January 2021, when COVID-19 imposed restrictions and rendered the healthcare system inoperative in the face of related weaknesses.

These “rare people” assisted by family members and caregivers had to follow the calendar drawn up by organizations attached to the Ministry of Health, promoting the questioning of families and support groups in defense of this population, who wondered why they were not given extreme priority in the vaccination program as a matter of fairness and intersectional social justice.

The need to understand the reasons and the resumption of the debate regarding the rights of rare disease carriers that, like the vast majority of Brazilians, are neglected by power holders and discredited by society, justifies this study, which seeks to substantiate the regular descriptors issued by official government bodies and by critics of rare disease issues in debates made in scientific articles available on the web.

The aim of this study is to conduct a critical analysis, focusing on the procedures of the National Immunization Program for the Brazilian population aged over 18, the care provided to individuals with rare diseases during the anti-COVID-19 vaccination campaign, and the evaluation of this plan’s effectiveness from the perspective of individuals with rare diseases in Brazil.

## 2. Materials and Methods

In order to conduct a comprehensive systematic documentary review of scientific and public health evidence, various data sources were employed, including academic databases, government official documents, technical reports, and the grey literature. Primary data sources encompassed PubMed, Scopus, Web of Science, Google Scholar, the Virtual Health Library (BVS), websites of the Ministry of Health of Brazil, and specialized organizations’ websites that are dedicated to rare disease patients. The search strategy was designed in accordance with the research objectives, entailing a combination of descriptors and relevant Boolean operators. Descriptors such as “COVID-19”, “vaccination”, “rare diseases”, “National Vaccination Plan”, “mutations”, and others in relation to research were used. The search strategy was adapted for each specific data source.

Inclusion criteria consisted of studies describing the evolution of COVID-19 from its discovery to subsequent mutations, including documents identifying procedures implemented by the National Immunization Program for the Brazilian population aged over 18 years, reports analyzing the care provided to rare disease patients during the Anti-COVID-19 vaccination campaign, and studies evaluating the effectiveness of this plan from the perspective of individuals with rare diseases. Exclusion criteria encompassed studies or documents unrelated to research objectives, duplicated publications, and works lacking full accessibility.

For selection from the literature, two independent reviewers conducted the initial screening of studies and documents based on the inclusion and exclusion criteria. Disagreements were resolved through a consensus, or in cases of persisting discrepancies, a third reviewer intervened. The quality of the included studies was assessed using specific criteria tailored to each study type. For non-academic documents, such as government reports, a credibility and relevance assessment was conducted by two independent reviewers. Relevant data from each study/document were extracted and organized into a database, incorporating information on disease evolution, vaccination procedures, care for patients with rare diseases, and an evaluation of vaccination plan efficacy.

Heterogeneity among studies/documents was evaluated, taking into consideration differences in study populations, methodologies, and outcomes. Substantial heterogeneity was addressed during qualitative analysis. The quality of evidence was assessed using the GRADE (Grading of Recommendations, Assessment, Development, and Evaluation) scale, considering criteria of quality and result consistency [1]. Regarding study limitations, non-academic documents may have limitations in terms of methodology and statistical analysis, which can influence the quality of evidence, and focus on the Brazilian perspective may limit the generalization of results.

## 3. COVID-19

COVID-19, the largest pandemic in recent human history, was caused by the novel coronavirus (SARS-CoV-2). It is a potentially severe acute respiratory infection with a global distribution that has high transmissibility via respiratory droplets or contact with contaminated objects and surfaces.

According to the World Health Organization (WHO), about 80% of people with COVID-19 recover from the disease without requiring hospital treatment. However, one in six people infected with SARS-CoV-2 become seriously ill and develop difficulties with breathing. The elderly and people with comorbidities, such as high blood pressure, heart and lung problems, diabetes, or cancer, have a higher risk of becoming seriously ill [2].

Since the initial genomic characterization of the SARS-CoV-2 virus, it has been divided into different genetic groups or clades, and when specific mutations occur, these can establish a new lineage (or genetic group) of the circulating virus.

It is also common to observe several processes of microevolution and virus selection pressures, and there may be some additional mutations that, as a result, generate differences within that lineage. When this happens, it is characterized as a new variant of the virus, and when mutations cause relevant clinical–epidemiological changes, such as greater severity and infectivity potential, this variant is classified as a VOC, in English, or variant of concern; in Portuguese, this is translated into a variant of attention and/or concern [3]. 

In Brazil, all of the VOCs described by WHO have already been identified and reported through laboratory and epidemiological monitoring established in the flow of health surveillance services.

As reported in the Epidemiological Bulletin number 73 [4], the SARS-CoV-2 virus, as well as other viruses, undergo expected mutations. To evaluate its genomic characterization in the network of the laboratory surveillance of respiratory viruses of MS, reference laboratories (Oswaldo Cruz Foundation—Fiocruz/RJ, Evandro Chagas Institute—IEC/PA and Adolfo Lutz Institute—IAL/SP) must be sent a flow of samples that are confirmed to be positive for COVID-19, via RTqPCR, which are sent for genomic sequencing and other complementary analyses, if necessary.

Additionally, in the period between 3 January and 10 July 2021, the epidemiological week (SE) ended on 27, 7545 records of COVID-19 cases observed for variants of care and/or concern (VOC) in the 27 FU of Brazil: 3 cases of VOC Beta (B.1.351) were identified in two municipalities of São Paulo; 27 cases of VOC Delta (B.1.617.2) were identified in seven federated units; 182 of VOC Alpha (B.1.1.7) were identified in fourteen federated units; and 7333 of VOC Gamma (P.1) were identified in all federated units, with VOC circulating predominantly in the country [4]. 

By the end of epidemiological week (SE) 29 in 2021, on 24 July 2021, 193,730,907 cases of COVID-19 had been confirmed worldwide. The United States was the country with the highest number of cumulative cases (34,428,050), followed by India (31,371,901), Brazil (19,670,534), France (6,041,146), and Russia (6,025,698)). In terms of deaths, 4,152,497 were confirmed worldwide until 24 July 2021. The United States was the country with the highest cumulative number of deaths (610,835), followed by Brazil (549,448), India (420,551), Mexico (238,316), and Peru (195,243) [4].

By the end of SE 29, according to Epidemiological Bulletin 73, 80.4% (128,107,515/159,302,857) of people infected with COVID-19 recovered worldwide, and data from the United States were ignored. India was the country with the highest number of recoveries (30,543,138 or 23.8%), followed by Brazil (18,340,760 or 14.3%), Turkey (5,415,937 or 4.2%), Russia (5,405,818 or 4.2%), and Argentina (4480 or 4333 or 2022%) [4].

Additionally, according to the Bulletin, in relation to deaths, on SE 29, 2021, Indonesia recorded the highest number of new deaths worldwide, reaching 9524 deaths. Brazil had the second-highest number of new deaths, reaching 8182 deaths. India recorded a total of 6942 new deaths, while Russia recorded 5361 new deaths, and South Africa recorded 2812, occupying the following positions in the world ranking of new deaths in SE 29.

## 4. Vaccination

The National Immunization Program (PNI), created on 18 September 1973, is responsible for the national immunization policy. Its mission is to reduce morbidity and mortality resulting from preventable diseases, strengthening integrated health surveillance actions for the protection of health and prevention in the Brazilian population. It is one of the largest vaccination programs in the world, recognized nationally and internationally. The PNI serves the entire Brazilian population, currently estimated at 211.8 million people. It is a moral patrimony of the Brazilian state, maintained by the commitment and dedication of health professionals, managers, and the entire population [2].

In order to collaborate in the preparation of this plan, the Ministry of Health established the Technical Advisory Chamber on Immunization and Communicable Diseases through the GAB/SVS Ordinance no. 28 of 3 September 2020, with the Coordination of the Secretariat of Health Surveillance, composed of representatives of this ministry and other governmental and non-governmental bodies, as well as scientific societies, class councils, specialists with expertise in the field, such as the Pan American Health Organization (PAHO), National Council of Health Secretaries (CONASS), and National Council of Municipal Health Secretariats (CONASEMS).

In the current scenario of great global health complexity, an effective and safe vaccine is recognized as a potential solution for controlling the pandemic, combined with the maintenance of preventative measures that have already been established.

Until 12 March 2021, WHO reported 182 COVID-19 vaccine candidates in the pre-clinical phase of research and 81 vaccine candidates in the clinical research phase. Of the vaccine candidates in clinical studies, 21 were involved in phase III of clinical trials for efficacy and safety evaluation: the last stage before approval by regulatory agencies and the subsequent immunization of the population [2].

Notably, the scope of people with comorbidities and people with permanent disabilities includes people with rare diseases, implying a greater risk for the unfavorable outcomes of COVID-19. For example, immunosuppressive diseases such as Cushing’s syndrome, systemic lupus erythematosus, Crohn’s disease, primary immunodeficiency with a predominance of antibody defects, and diseases that cause chronic lung impairment such as cystic fibrosis are cited, as are diseases that cause intellectual and/or motor and cognitive disabilities such as Cornelia de Lange syndrome, and Huntington’s disease, and other rare diseases such as sickle cell anemia and thalassemia major [3].

According to Technical Note 467/2021, the definition of priority groups for vaccination was based on epidemiological analyses and evidence and discussions with experts on immunization and the main scientific societies within the Technical Advisory Chamber on Immunization and Communicable Diseases. It was also based on the recommendations of SAGE—Strategic Advisor Group of Experts on Immunizing (Immunizing)—of the World Health Organization, in partnership with Tripartite, with the National Councils of Health Secretaries and Municipal Health Secretariats (Conass and Conasems) [3].

Additionally, according to NT 467, given the still-limited availability of vaccines to offer to the target population of the National Vaccination Campaign against COVID-19 2021, PNI ratified the importance of those made available to predetermined groups, which were at higher risk of exposure, complications, and death from COVID-19, according to the priorities listed in the National Plan for the Operationalization of Vaccination against COVID-19 (PNO).

According to Alexandre Padilha, a member of the Committee on the Rights of Persons with Disabilities of the Chamber of Deputies of Brazil, rare disease sufferers in Brazil have vulnerabilities that proceed beyond their clinical and epidemiological situations. The criterion of vaccination priority in the country cannot be a “cut and glue” of the reality of other countries, where social inequality is lower [5].

According to Padilha, the definition of a priority group for any vaccination campaign considers two factors: clinical or epidemiological vulnerability. These indicators show that a person may have a more severe risk of an illness or other vulnerabilities.

According to [2], people with rare diseases and at greater risk of coronavirus are included in the group of comorbidities and permanent disabilities. Among these diseases are those that cause immunosuppression, such as Cushing syndrome, systemic lupus erythematosus, and primary immunodeficiency with the predominance of antibody defects; diseases that cause chronic pulmonary impairment, such as cystic fibrosis, sickle cell anemia, and thalassemia major; and syndromes that cause intellectual disability, such as Cornelia de Lange.

The analysis of vaccination coverage by age groups in the population aged 18 and over by the federative unit (FU) and based on complete vaccination schedule data (D2 for Sinovac/Butantan and Covishield/AstraZeneca/Fiocruz and Pfizer) and DU for the Janssen vaccine, showed different scenarios in different groups and FU [4].

According to the Epidemiological Bulletinnumber 73, the vaccination coverage (CV) national average in each group was below 15%, with variations between just over 2% in the 18–19-year-old age group and 14% in the groups from 40 to 44 and 45 to 49 years of age.

## 5. Vaccines

According to the Ministry of Healththe COVID-19 vaccines distributed for use so far in the National Campaign are as follows [4]:

(a) Butantan Institute (IB): vaccine adsorbed COVID-19 (Inactivated). Producer: Sinovac Life Sciences Co., Ltd. (Singapore). Partnership: Sinovac/Butantan.
(b) Oswaldo Cruz Foundation—Institute of Technology in Immunobiologicals—BioManguinhos (Fiocruz/BioManguinhos): COVID-19 vaccine (recombinant) Manufacturer: Serum Institute of India Pvt. Ltd. (Bangalore, India). Partnership: AstraZeneca/Fiocruz.
(c) AstraZeneca: vaccine against COVID-19 (ChAdOx1-S (recombinant). Vaccine from the Covax Facility consortium.
(d) Pfizer/Wyeth: COVID-19 vaccine (mRNA) (Comirnaty)—Pfizer/Wyeth.
(e) Janssen: COVID-19 vaccine (recombinant). Vaccine from the Covax Facility consortium.

Up until the 30th Technical Report of 26 July 2021, 32 vaccine distribution guidelines were implemented, enabling the delivery of approximately 168 million doses, with approximately 84.2 million doses of AstraZeneca/Fiocruz vaccine; 59 million doses of Sinovac/Butantan vaccine; 19.9 million of the Pfizer/Comirnaty vaccine; and 4.5 million doses of the Janssen vaccine (Johnson & Johnson), reaching approximately 92.7 million people vaccinated [4].

According to the Epidemiological Bulletin number 73, at least three of the four COVID-19 vaccines available in the country can be administered in two doses to ensure greater effectiveness [4].

Our study continues:

In collaboration with experts from its network of institutions and research worldwide, the World Health Organization (WHO) routinely evaluates variants of the SARS-CoV-2 virus. These analyses mainly observe whether the behavior of new variants results in changes to transmissibility, in the disease clinic and also in its severity; some changes may suggest decision-making and the implementation of new measures for the prevention and control of the disease. An established and timely genomic surveillance contributes to the strengthening of such guidelines, and in the current pandemic scenario, this guides managers’ decision making [4].

Based on these analyses, booster doses and an expansion of the target groups for vaccination are already planned.

## 6. Rare Disorders

There are approximately 7000 diseases worldwide that are currently considered to be rare; however, there is no universal concept for the term “rare disease”, which refers to an illness characterized by its reduced appearance in the population [6,7]. 

In most countries, to classify a disease as rare, a cutoff number in the prevalence rate is determined. In the European Union (EU), rare diseases are considered potentially fatal or chronically debilitating, with a prevalence lower than 5:10,000, and they require combined efforts between various areas to prevent significant morbidity and mortality or a considerable reduction in quality of life or the socioeconomic potential of the patient. The number of affected patients in the EU is estimated to be close to 36 million. In the United States, the Orphan Drug Act of 1983 defines rare disease as any disease or condition that affects less than 200,000 people in the country. In Japan, by the definition of the Japanese Medicines Act of 1993, a rare disease cannot affect more than 50,000 people in the country [8].

In Ordinance 199 of 30 January 2014, in its third article, the Ministry of Health defined a rare disease as one that has an incidence lower than 1.3 per 2000 inhabitants and afflicts up to 65 people out of 100,000 individuals. In other words, it is a disease that affects less than 5% of the population in a given territory [9].

According to the Brazilian Federation of Rare Disease Associations—Febrararas)—people living with rare diseases have, in general, chronic and multisystemic conditions, which place them in a risk group with greater physical and psychosocial vulnerability. There are many rare diseases, and they are diverse in their etiologies, signs, symptoms, and treatments. But every rare disease has something in common: it affects people. This may seem obvious, but people with rare diseases suffer as a result of the lack of scientific and medical knowledge about their health conditions [10].

Studies in relation to rare diseases have attracted increasing recognition in the social context. One of the possible explanations for this growth is related to the impact of civil associations of rare diseases on society; for instance, a breakthrough in genetic research culminated in the discovery of treatments for diseases that were previously considered incurable [11].

COVID-19, the disease responsible for the largest pandemic of the 21st century, has affected people of all ages, and its signs and symptoms can vary according to age and clinical conditions. Age has a great influence on the individual risk of influenza, with a higher incidence in young people and more expressive lethality in the elderly and in individuals with clinical conditions or comorbidities that can put them at risk of influenza complications. These complications include the exacerbation of chronic conditions and other serious complications such as bacterial infection, myocarditis, pericarditis, bronchiolitis, and encephalitis [12]. For the rare disease carrier, there have been few specific reports on coronavirus infection, although there are specific groups of patients who may be at greater risk due to a secondary condition (disease) or a complication of his/her inherited metabolic disease. These include the following [10]:

(a) People with chronic lung or heart disease (this may, for example, include some patients with mucopolysaccharidosis, mitochondrial disease, or Pompe disease).
(b) People with muscular dystrophies as they usually have heart and lung involvement.
(c) People with diabetes or a metabolic condition.
(d) Those taking immunosuppressive drugs, for example, after a transplant, may have a higher risk of complications if they are affected by coronavirus, e.g., metabolic diseases with concomitant neutropenia.
(e) Some people with inherited metabolic disease are at risk of worsening (decompensation) their metabolic condition, which may lead to the development of a viral infection.
(f) Any patient requiring an emergency regimen, including individuals with defects in the urea cycle, fatty acid oxidation disorders, maple syrup urine disease, methylmalonic acidemia, glutaric aciduria type 1, or Propylonic acidemia, must have specific supplements or medicines to keep at home for use in case of illness and metabolic decompensation.
(g) Patients with neuromuscular disorders, such as Duchenne Muscular Dystrophy, who have impaired ventilation or are using corticosteroids.
(h) Patients with airway malformations.

According to Regina Próspero, in an interview with Portal Kangaroo News (2021), it was estimated that there are about 15 million people with rare diseases in Brazil. Despite this significant number of “rare people”, there is still widespread ignorance about the subject in society and even among the medical community, which often results in difficulties during the diagnosis, treatment, and monitoring of conditions.

## 7. Analysis

The coronavirus surprised the world with its speed and lethality, causing concern, despair, and the union of the scientific and political communities around the world because people were dying.

The process of investigating this disease has led scientists from all over the world to work intensively in research and testing and to discuss health in all its aspects, especially structural, whose weaknesses were evident before the health emergency in several countries.

In this sense, according to the World Health Organization, the detection and spread of an emerging respiratory pathogen are accompanied by uncertainty about its epidemiological, clinical, and viral characteristics, particularly its ability to spread among the human population and its virulence (case–severity) [4].

It has been stated that “in view of this, the pandemic resulting from human infection by the new coronavirus has caused impacts with global losses of social and economic order, becoming the greatest public health challenge”.

The issues resulting from the COVID-19 pandemic highlighted those who were underprivileged by health systems, including those with rare diseases that, according to the material identified in this research, were not referenced in the vaccination process since they were included in the comorbidities group.

On an official website, Marina Pagno reported that the Ministry of Health “defined a strategy for vaccination against COVID-19 for people with permanent disabilities, pregnant women, postpartum women up to 45 days postpartum, and people with comorbidities, including some rare diseases”.

Based on this plan, according to Pagno, vaccination occurred in two stages that, although encompassing people with general comorbidities, did not favor “rare people”. He stated the following [2]:

In phase 1, the following shall be vaccinated:

- People with Down syndrome, regardless of age.
- People with chronic kidney disease who are on renal replacement therapy (dialysis), regardless of age.
- Pregnant women and postpartum women with comorbidities, regardless of age.
- People with comorbidities aged 55 to 59.
- People with permanent disabilities registered in the Continuing Benefit Program (BPC) aged 55 to 59 years.

Phase 2, on the other hand, should consider older to younger individuals (50 to 54 years, 45 to 49 years, 40 to 44 years, 30 to 39 years, and 18 to 29 years):

- People with comorbidities.
- People with permanent disabilities registered with the BPC.
- Pregnant and puerperal women, regardless of pre-existing conditions.

Rare disease patients require special (often constant) care and specific, expensive medicines, and orphans are part of the routine of families caring for “rare people”.

As a result of these considerations, families questioned the inconsistency of the National Vaccination Plan, including those with rare diseases in the category “comorbidities” and family members, usually their caregivers, in the general category when the respective age groups were contemplated.

Thus, in a Public Hearing promoted by the Commission for the Defense of the Rights of Persons with Disabilities—CPD—on 8 June 2021, Mrs. Andréa Medrado Monteiro raised the following question:

(A) Why, in some states, such as Rio de Janeiro, is the vaccination of caregivers of people with rare diseases happening, and in the other states, it is not? Why not expand to all of Brazil as a priority group? After all, if there are more than 13 million people with rare diseases in the country, imagine how many parents there are.

The Vaccine Already PCD and Rare Diseases Movement posed the following questions and observations:

(B) People with rare diseases are dying, and this segment does not have time for your evaluation and possible full PNI contemplation. We need vaccines now! The at-risk groups include children and their caregivers, usually parents who go to work and expose themselves to the virus. When will these caregivers/parents be in the PNI?
(C) Communication is everything! State-of-the-art health workers are not vaccinating the few that are contemplated, for example, those with neurological diseases. They refuse to vaccinate patients with rare diseases due to an ignorance of the diseases in question.

Mrs. Raquel Carvalho Pinheiro, identifying herself as being at risk, raised the following question:

(D) Hello! I am a caregiver of a child with a rare syndrome, and we need to be vaccinated. Who will take care of our children if we get sick? Moreover, they are in an at-risk group if contaminated. Vaccinating people with rare diseases and syndromes and their caregivers is an act of humanity. Additionally, I am talking about the vaccine for domestic caregivers. The reality of families is not having professional caregivers. Look at this reality, please.

The reality of families is delicate because there is no guarantee of a cure or one hundred percent immunity to the disease even with the application of available vaccines, especially considering the spread of variants from other continents, the prevalence of the Delta Variant, and the greater power of contagion and lethality.

This reality is consistent with the statement of the PNI:

In collaboration with experts from its network of institutions and research worldwide, the World Health Organization (WHO) routinely evaluates SARS-CoV-2 virus variants. This genomic sequencing analysis mainly observes whether the behavior of new variants has resulted in changes in transmissibility in the clinic of the disease, in severity, and also in the vaccine response; some changes may suggest the decision making of national authorities to implement new measures for the prevention and control of the disease. Established and timely genomic surveillance collaborates to strengthen such guidelines, and during the current pandemic scenario, this is a tool that guides managers’ decision making [4].

The worsening of the COVID-19 pandemic due to variants, which are common in flu syndromes, not only threatens “rare patients” with greater fragility but also those who are already participating in the vaccination program because immunizers experience, according to experts, relief from the symptoms and aggravations caused by SARS-CoV-2.

When discussing public health in the current scenario, one must refer back to management, which undoubtedly has power over the planning, procurement, operation, and control of processes, registration, validation, and dissemination of results.

The stigmatized “rare person” remains unseen and does not receive the attention of their society; this extends to their families.

These issues have recently been acknowledged by governmental actions and non-governmental organizations, and some achievements have been celebrated; however, these people must fight for their rights with the judiciary more often than desired.

Even in the face of this frightening scenario, we cannot lose the fighting horizon. No one can think that we will be protected by ensuring individual vaccination. We will be safe only with collective vaccination. And, even if we have a vaccination for everyone, it is important that vaccination is still a priority. These two groups and their caregivers should be in the priority population [5].

To this end, the immunization program must be continuous, but with the desire that these unprivileged and their close ones are actually considered priority members in this process of life preservation.

## 8. Final Remarks

The new coronavirus highlighted the strength, importance, and power of science, which managed to map the genome of this virus in record time, identify drugs intended for palliative treatment, and develop various immunizations that have met the expectations of states and nations.

Given the numerous mutations of this virus, the effectiveness of vaccines has been investigated, considering the application of a booster dose for the priority groups determined by the National Vaccination Plan, in which rare disease patients and family members are still not prioritized, the former being assigned to the group of general comorbidities and the others to the order determined by their age group.

It is worth mentioning, in this analysis, that the most recent data date back to July 2021, when groups composed of age groups from 18 to 45 years had not yet been considered eligible for the initial doses of the vaccine: not even a single dose of the pharmaceutical Johnson & Johnson.

As the Ministry of Health recalls regarding vaccination:

Its main objective was defined as priority, the preservation of the functioning of health services; the protection of individuals at greater risk of developing severe forms of the disease; the protection of other vulnerable individuals to the greatest impacts of the pandemic; followed by the preservation of the functioning of essential services [4].

Thus, with SARS-CoV-2 variants affecting all continents, it is necessary to expand the immunization campaign with the application of reinforcement; acquisition, upon approval by a corresponding body (in the case of ANVISA), of new immunizers; expansion to groups aged under 18; and to reconsider, as soon as possible, the protocol for vaccinating “rare people”, who should be an exceptional priority, since their weaknesses become evident more quickly.

Efforts must still be made to disseminate information safely and effectively so that the objectives of the PNI are achieved, at least in their minimum coverage expectations; it is believed that this population is aware of the importance of immunizations and of compliance with the current safety protocols.

As for rare disease patients, managers must pay greater attention to the group that exists and resists in this family, which is neglected even by health agents.
